# Prophylactic and Early Thyroidectomy in *RET* Germline Mutation Carriers in Pediatric and Adult Population: Long-Term Outcomes of a Series of 63 Patients

**DOI:** 10.3390/cancers14246226

**Published:** 2022-12-17

**Authors:** Francesca Torresan, Simona Censi, Gianmaria Pennelli, Francesca Galuppini, Caterina Mian, Maurizio Iacobone

**Affiliations:** 1 Endocrine Surgery Unit, Department of Surgery, Oncology and Gastroenterology, University of Padova, Via Giustiniani 2, 35128 Padova, Italy; 2 Endocrinology Unit, Department of Medicine, University of Padova, 35128 Padova, Italy; 3 Pathology Unit, Department of Medicine, University of Padova, 35128 Padova, Italy

**Keywords:** pediatric population, adult population, hereditary medullary thyroid carcinoma, C-cells hyperplasia, prophylactic thyroidectomy, *RET* mutations

## Abstract

**Simple Summary:**

Approximately 20% of medullary thyroid cancers (MTC) are hereditary and caused by specific germline *RET* protooncogene mutations. Screening for germline mutation in all newly identified cases and their relatives allows early or even prophylactic surgical treatment. The aim of this retrospective study was to assess the clinical features of *RET* mutation carriers according to the age at surgery and the long-term outcome in term of morbidity and recurrence rate after prophylactic and early thyroidectomy. The results demonstrated that prophylactic and early thyroidectomy are safe and effective procedures in achieving the cure; however, while patients with only C-cell hyperplasia are disease-free, patients with MTC, even when small and confined to the gland, could have recurrent disease at long-term. This finding suggest that prophylactic surgery should be performed earlier.

**Abstract:**

Prophylactic and early thyroidectomy in *RET* germline mutation carriers allows the removal of the thyroid before medullary thyroid carcinoma (MTC) develops, or while it is still confined to the gland. This study was aimed to assess the clinicopathological features in *RET* carriers according to the age at surgery and the long-term outcomes after prophylactic and early thyroidectomy. A retrospective analysis of 63 operated asymptomatic *RET* carriers diagnosed after familial genetic screening was performed. Twenty-one *RET* carriers were operated at pediatric (<18 yrs) and 42 at adult (≥18 yrs) age. Serum preoperative calcitonin levels were significantly lower in pediatric compared to adult patients (*p* = 0.04); moreover, adult *RET* carriers had a greater frequency of microMTC at pathology (*p* = 0.009). Permanent postoperative morbidity occurred in 9.5% of patients, without differences between the two groups. Biochemical postoperative cure was achieved in all patients. At a median follow-up of 14 years, all C-cell hyperplasia patients are disease-free; conversely, biochemical, and structural recurrence of disease occurred in three adults and one pediatric patient with microMTC. The independent predictive factors of MTC were the age at surgery, the preoperative calcitonin level and the *RET* mutational risk profile (*p* < 0.02). In conclusion, prophylactic and early thyroidectomy are safe and effective procedures in achieving definitive cure in most *RET* carriers. However, since recurrences may occur at long-term in case of microMTC, thyroidectomy should be possibly performed earlier to prevent microMTC development.

## 1. Introduction

Medullary thyroid carcinoma (MTC) is a rare calcitonin (Ct)-secreting neuroendocrine tumor that arises from parafollicular C-cells. It may occur as an autosomal dominant hereditary disease in approximately 20% of cases, as part of the multiple endocrine neoplasia type 2 (MEN2) syndrome caused by *RET* protooncogene germline mutation. Several mutations have been described with different clinical expression and variable penetrance; according to the American Thyroid Association (ATA) classification [1], patients carrying a high (H)-risk mutation are predisposed to develop MTC at an earlier age and should be operated before 5 years of age. Conversely, moderate (MOD)-risk mutation may cause MTC development later, even in adulthood. The genetic screening in all relatives of newly diagnosed MTC cases is aimed to the identification of asymptomatic and apparently healthy *RET* carriers allowing a definitive cure by a prophylactic thyroidectomy, removing the thyroid before malignancy occurs (as usually occurs in children), or by an early thyroidectomy when MTC is still confined to the gland (as usually may occur in adults) [1,2,3].

However, the optimal age and the extent of surgery in *RET* carriers is still debated, with some authors claiming in favor of Ct dosage and some authors in favor of a genomic-based approach [4]. Moreover, in common clinical practice, a tendency to postpone surgery in children may occur because total thyroidectomy is a challenging procedure with increased morbidity when performed at pediatric age [5], or following parental preference. The long-term outcome in terms of cure and surgical morbidity following prophylactic or early thyroidectomy has been poorly evaluated in apparently healthy *RET* carriers, especially in relation to the age at surgery, histology, and mutational risk profile.

The aim of this study was to assess the clinicopathological features in apparently healthy and asymptomatic *RET* mutation carriers according to the age at surgery and ATA risk mutation, and the long-term outcomes after prophylactic and early thyroidectomy.

## 2. Materials and Methods

The present retrospective study included a series of 63 asymptomatic and apparently healthy *RET* mutation carriers diagnosed after family screening, undergoing prophylactic or early thyroidectomy at the Endocrine Surgery Unit of Padova University Hospital, Italy, between 1986 and 2021.

Prophylactic and early thyroidectomy were retrospectively defined as the removal of the thyroid before MTC developed or while it was clinically unapparent and confined to the gland, respectively. The study included: (i) all clinically apparently healthy *RET* carriers’ patients newly diagnosed by *RET* genetic screening or Ct dosage for family history; (ii) without clinical signs or symptoms of MTC; and (iii) without the suspicion of thyroid malignancy at neck ultrasound (US) or fine-needle aspiration cytology, or at intraoperative gross examination that iii) underwent at least total thyroidectomy. Patients with a pre- or intra-operative diagnosis of MTC were excluded.

Before undergoing genetic testing, all patients or their parents gave informed consent. For identification of RET mutations, genomic DNA was purified from peripheral blood leukocytes and then amplified using PCR. The *RET* gene was analyzed by direct sequencing of exons 5, 8, 10, 11, 13, 14, 15, 16. *RET* genetic analysis was performed in all patients with positive family history after 2000, with the discovery of the pathogenetic variants. Before 2000, when *RET* gene analysis was not even or systematically available, Ct dosage and neck US have been used to screen patients with family history of MTC, as previously stated [6]. In this latter scenario, the diagnosis was later definitely confirmed by genetic analysis in all cases.

Patients were classified as pediatric or adult population if operated at an age <18 or ≥18 years, respectively. Patients were classified as ATA moderate (MOD) or high (H) risk according to the 2015 revised ATA practice guidelines [1]. In- and out-patient medical records were reviewed to gather relevant demographics (gender, age at surgery), clinical features, pre- and postoperative Ct values, surgical treatment, and pathology finding. Follow-up data included biochemical, clinical, and radiological evaluation.

Postoperative outcomes and follow-up data were assessed according to the last available medical record. Only patients with complete outcome follow-up data (at least 6 months) were included. The preoperative workup included Ct measurement, neck US and fine needle aspiration cytology in case of nodules, to exclude the presence of a clinically evident MTC; and calcium, parathormone (PTH), 24-h urine catecholamines and metanephrines measurements to assess associated multiple endocrine neoplasms.

Since different methods have been used to measure Ct, with no comparable sensitivity, specificity and normal ranges, the Ct levels were normalized and scored as a ratio (Ct value/upper limit of the normal range value), as previously described [6,7]. All patients underwent total thyroidectomy (TT); central neck lymph node removal (CNR) at various extent was also performed in some cases. The follow-up evaluation was systematically performed and included Ct, calcium and PTH dosage, neck US, computed tomography or magnetic resonance assessment. The end points of the study were the outcomes in terms of cure or persistent/recurrent disease, as assessed at the time of the last available follow-up, and postoperative morbidity (rate of transient or persistent hypoparathyroidism and vocal cord paralysis). Postoperative hypoparathyroidism was defined as the presence of serum calcium levels below the normal values (total serum calcium < 2.00 mmol/L) with inappropriately low PTH levels [8]. Vocal cord mobility was determined by direct laryngoscopy. Morbidity persisting for more than 6 months after surgery was considered permanent.

Cure was defined as a postoperative disease-free status (undetectable serum Ct levels in the absence of clinical and radiological signs of the disease). Persistent and recurrent MTC (biochemical, clinical and/or radiological evidence of disease) were defined by the occurrence of disease following a postoperative cure < or >6 months, respectively. Biochemical persistence/recurrence of disease was defined as the presence of detectable serum Ct levels exceeding the normal upper values; structural relapse was defined by the presence of clinical or radiological signs of MTC.

The study was performed in accordance with the guidelines in the Declaration of Helsinki and was approved by the Institution Review Board. Informed consent was obtained from all subjects involved in the study. Data were expressed as absolute numbers, median and interquartile range (IQR), range or 95% confidence interval (CI). Student’s t test, Mann–Whitney, Chi-square, Fisher’s exact test were used, as appropriate. Ct ratio levels and age were analyzed as continuous and categorical variables (Ct < 1 and Ct ≥ 1; <18 years and ≥18 years, respectively). Receiver operating characteristic (ROC) curve analysis was used to identify a Ct threshold of increased risk of medullary thyroid carcinoma, calculating the area under the ROC curve (AUC) and sensitivity and specificity at Youden’s index. Multivariable analysis was conducted by the logistic regression analysis. Recurrence was calculated as the time from surgery until the time of disease relapse and analyzed by the Kaplan–Meier methods and the log-rank test. *p* value <0.05 was considered statistically significant.

## 3. Results

Sixty-three MEN 2A gene carriers (39 female and 24 male), aged from 5 to 70 years (median 25), from 39 kindreds, were included in the study (Table 1).

The median preoperative Ct ratio was 1.02 (range 0.05 to 43). Normal serum Ct levels (Ct levels ratio < 1) were found in 31 patients (median age 19, range 5–70 years), while 32 had elevated serum basal preoperative Ct concentrations (median age 29, range 5–63 years old). No significant differences in serum Ct concentration between male and female patients were found.

**Figure 1:**
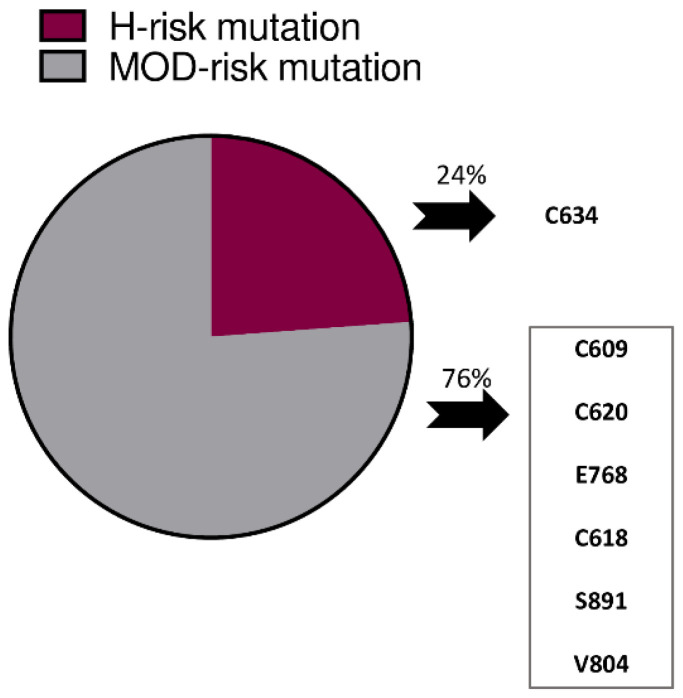
*RET* mutations in 63 patients undergoing prophylactic or early thyroidectomy.

The *RET* mutations are reported in Figure 1. The majority of the patients (76%) had an ATA MOD-risk mutation; the remaining 24% were ATA H-risk mutation carriers in codon 634. Total thyroidectomy was performed in all patients; lymph-nodal status was available in 57% of cases (67% of adults and 73% of H-risk patients) and showed no nodal metastases in all cases at pathology. Patients undergoing total thyroidectomy and nodal removal had significantly greater serum Ct levels compared to those undergoing only total thyroidectomy (1.4 vs. 0.6, respectively; *p* = 0.003). MTC was found at pathology in 70% of the *RET* carriers, with a median size of the greatest lesion of 4 mm (range 1–17 mm). It was multifocal in 27 patients, and also bilateral in 16 cases. The size of the largest tumor was <10 mm in the vast majority (40 out of 44 patients, 91%). In the remaining 19 patients, only C-cell hyperplasia was found.

**Figure 2:**
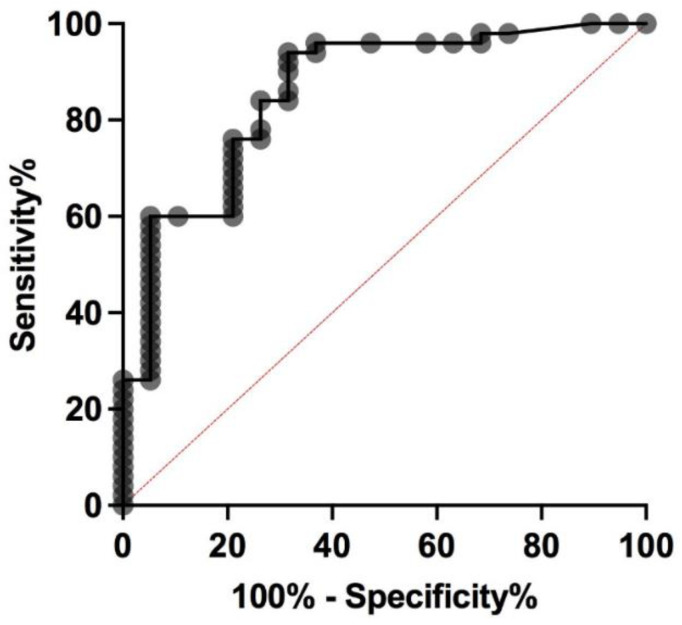
ROC curve showing specificity and sensitivity of preoperative serum calcitonin levels in the detection of medullary thyroid carcinoma in 63 *RET* mutations carriers undergoing thyroidectomy.

ROC curve analysis showed that the sensitivity and specificity of preoperative serum Ct levels (expressed as a ratio) to detect an MTC were 93.2% and 68.4%, respectively, using the identified Youden index cut off of 0.5 (Figure 2). Moreover, no significant correlation was found between Ct levels and tumor size (r^2^ = 0.2; *p* = ns).

### 3.1. Clinical, Genetic and Pathological Findings According to the Age at Surgery

Twenty-one *RET* mutation carriers were operated in pediatric and 42 in adult age. In pediatric population, 57% of the patients were younger than 12 years. No differences in term of gender and *RET* risk mutational profile were found between the two population (Table 2). Younger patients had a lower risk of MTC compared to adults (48% vs. 81%, respectively; *p* = 0.009), smaller tumors (2 mm vs. 5 mm, *p* = 0.0016) and lower preoperative Ct values (0.6 vs. 1.15; *p* = 0.04); moreover, a trend toward normal preoperative Ct values (measured as ratio <1; *p* = 0.06), and less extensive surgery (with CNR in 38% versus 67%; *p* = 0.057) were found.

### 3.2. Clinical, Genetic and Pathological Findings According to the ATA Risk Mutational Profile

A significant relationship between the ATA *RET* risk mutation category and the stage of the C-cell disease was detected (Table 3). In H-risk *RET* mutation patients, a significant increased risk of MTC was found compared to MOD-risk category (93% versus 62.5%; *p* = 0.026). No differences regarding gender, age at surgery, Ct levels and extent of surgery was found between the two groups. H-risk patients underwent surgery at a median age of 18 (range 5–46); 7 patients underwent surgery at pediatric age (median age 10 years, range 5–17) and 8 in adulthood (median age 25, range 18–46). MTC was detected in all patients, except a case of CCH detected at the age of 25 years. In MOD-risk patients, surgery was performed at pediatric age in 14 patients (median age 13, range 6–17) and in 34 patients in adulthood (median age 32, range 18–70). MTC was found in 3 patients in pediatric age vs. 27 in adulthood (21% vs. 79%; *p* = 0.0003).

### 3.3. Surgical Morbidity According to Age at Surgery and Extent of Surgery

Morbidity occurred in 20 patients, without differences between pediatric and adult population (33% vs. 31%, respectively) (Table 4). Transient postoperative hypoparathyroidism occurred in 18 patients, without significant differences between pediatric and adult patients (33% vs. 26%, respectively; *p* = 0.57). Transient vocal cord paresis occurred in four patients, without statistical differences between the two groups. Permanent postoperative morbidity occurred in 9.5% of cases (hypoparathyroidism in four cases; unilateral vocal cord paresis in two), without any statistical difference between the two groups. Definitive hypoparathyroidism occurred only in adult patients. Permanent unilateral cord paresis occurred in two patients (one child and one adult), undergoing TT + CNR.

### 3.4. Predictive Factors of MTC

At uni- and multivariable analysis, the mutational risk profile (OR 18.5), the age at surgery (OR 7.51) and the preoperative Ct level ratio (OR 5.02) were the main predictors of MTC (*p* < 0.02) (Table 5).

### 3.5. Follow-Up

Early biochemical postoperative cure was achieved in all patients.

**Figure 3:**
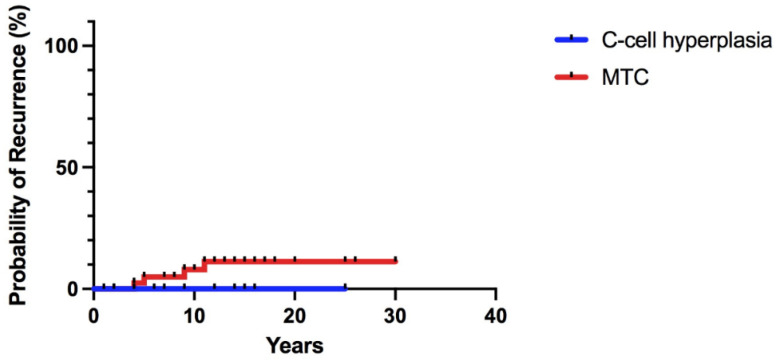
Recurrence probability in 63 RET mutations carriers with C-cell hyperplasia and medullary thyroid carcinoma (MTC).

At a median follow-up of 14 years (range 6 months–36 years), even if no statistically significant differences were found, all patients with C-cell hyperplasia are disease-free; conversely, four patients (three adults and one children) with a microMTC undergoing TT + CNR developed a biochemical relapse; one of them had also a structural relapse 13 years after initial surgery (Figure 3). No mortality due to MTC-related causes was observed.

## 4. Discussion

Systematic *RET* genetic testing of relatives in MTC index cases allows the early detection of healthy germline *RET* mutation carriers; thus, these patients can be offered a prophylactic or early thyroidectomy, achieving a definitive cure of the disease, preventing the risk of late consequences of MTC [9]. Over the past 25 years, a relevant number of studies have highlighted a close genotype-phenotype correlation in MEN2 gene carriers. The 2015 revised American guidelines for the management of MTC have reclassified the risk profile of mutations in highest, high, and moderate risk categories [1], suggesting the optimal time for surgery in *RET* carrier children. However, the timing of thyroidectomy in children remains debated, and the benefits of prophylactic or early thyroidectomy in term of cure should be balanced against the surgical morbidity that is notoriously increased in children. 

The present study describes the clinicopathological features and surgical outcomes of a cohort of 63 asymptomatic *RET* mutation carriers undergoing prophylactic or early thyroidectomy during pediatric or adult age. As previously reported [1], a close correlation between the age and the risk of C-cell transformation has been found. In the present cohort of asymptomatic *RET* mutation carriers, patients older than 18 years at the time of surgery had a significantly greater probability to present MTC at histology compared to children. However, a prophylactic or early thyroidectomy is also possible during adult age; in fact, only CCH was found at histology in a not negligible proportion (19%) of adult patients; thus, a “truly” prophylactic TT can be obtained even in patients older than 18.

Moreover, as suggested by multiple series [1,10,11,12], the age-dependent progression from CCH to MTC can be grouped according to the genetic risk profile. In the present series, independently from age at surgery, asymptomatic H-risk *RET* mutation carriers had a significant greater risk to harbor an MTC compared to those patients carrying a MOD-risk *RET* mutation (93% vs. 62.5%). According to the guidelines [1], the *RET* mutation risk category, reflecting the clinical aggressiveness of the corresponding MTC and its correlated age-dependent development, should be currently used to plan the optimal timing of prophylactic thyroidectomy. H-risk patients should possibly undergo surgery at the age of 5 years or earlier; in the present series, the majority of H-risk *RET* carriers underwent surgery later, due to the late discovery of *RET* mutation in the index case, or the parents’ refusal of surgery at a very early age. As a consequence, MTC was found in almost all H-risk cases (93%). Nevertheless, controversies about the age in which TT should be performed still exists, in particular for the MOD-risk *RET* mutations. Moreover, there are often concerns about the impact on the quality of life of surgery performed in children, due to physical and psychological traumatism, and the adherence to hormonal substitutive treatment, leading sometimes the parents to postpone the timing of thyroidectomy.

Some authors have suggested that the genetic data alone do not allow an accurate prediction of C-cells transformation and then suggested to use the biochemical Ct screening to define the optimal time for surgery, and to guide the extent of surgery. It has been suggested that TT in *RET* carriers can be delayed beyond 5 years old if there is no evidence of suspected lymph nodes, serum Ct is below 40 pg/mL and if only micronodules < 5 mm are found at neck US [10,12,13,14]. Elisei et al. suggested that in *RET* mutation carriers with undetectable serum Ct levels the time of TT can be personalized and safely planned when basal and/or stimulated Ct becomes positive, independently of the type of *RET* mutation and its associated level of risk [4]. However, based on the present experience, even serum Ct levels did not reach a 100% sensitivity and specificity in identifying asymptomatic *RET* carriers with MTC. In fact, as recently pointed out, even if Ct dosage is the most accurate way to detect MTC, Ct assay suffers from several pre-analytical, analytical and postanalytical pitfalls; for these reasons, proCt has been recently suggested as a potential marker of MTC, but no data in hereditary MTC are available [15].

The morbidity associated with prophylactic and early TT was found not negligible in children. In particular, the rate of hypoparathyroidism has been reported to be significantly higher in children compared to adults, due to the smaller size and translucency of the parathyroid glands, that are difficult to differentiate from the adjacent structures and soft tissues [16,17]. Based on the present experience, no significant increase of transient and permanent morbidity between patients operated during the childhood or adulthood, has been found. Even if possibly biased by the relatively small sample of the series, and by the retrospective design of the study and the involvement of different surgeons overtime, this is in line with other more recent experiences from high volume specialized centers [18,19].

In the present series, the early biochemical cure was obtained in all patients; after a median follow-up of 14 years, a permanent disease-free status occurred in patients with CCH (100%); conversely, four patients (one child and three adults) with MTC (9.1%) developed a biochemical relapse; one of them had a clinically detectable recurrence 13 years after initial surgery. Therefore, patients with microMTC, independently of the age at surgery, after an early thyroidectomy require a life-long follow-up, since recurrences can occur even at long-term interval.

Based on the present experience, the independent predictors of MTC were the *RET* risk mutational profile, the age at surgery, and the Ct levels. Therefore, *RET* mutation carriers should be operated as soon as possible at a young age, especially those carrying a H-risk *RET* mutation, and when Ct levels are within normal range, to avoid once and for all the risk of developing MTC and a possible relapse of the disease that may occur even at long-term. The morbidity associated with prophylactic thyroidectomy, possibly avoiding a central lymph nodes dissection in children, might be acceptable if performed in experienced hands.

Limitations to be acknowledged in this study include its retrospective nature, the relatively small number of included patients that preclude further sub-analysis, the lack of a standardize surgical approach, and the presence of different surgeons and pathologists, even if expert in thyroid surgery, over a long time period. However, even with these limitations and considering the rarity of the disease, the results of the present study contribute to expand the current knowledge about the outcome of prophylactic and early thyroidectomy in asymptomatic *RET* carriers in a monocentric series from a high-volume referral center.

## 5. Conclusions

Even if limited by the retrospective nature of the study and the relatively small sample size, prophylactic and early TT seem to be safe and effective in achieving definitive cure even in asymptomatic adult *RET* mutation carriers. However, since recurrences may occur even at long-term follow-up in case of microMTC, surgery should be performed earlier at pediatric age to prevent microMTC development, and to obtain definitive long-term cure with acceptable morbidity.

## Figures and Tables

**Table 1:** Clinical and pathological findings of the 63 thyroidectomized patients undergoing prophylactic or early thyroidectomy.

Characteristics		n = (%)
Age (years)	<18	21 (33%)
	≥18	42 (67%)
Sex (F/M ratio)		1.6
Pre-op Ct levels		1.02 (1.8)
*RET* mutation	MOD-risk	48 (76%)
	H-risk	15 (24%)
Surgery	TT	27 (43%)
	TT + CNR	36 (57%)
Pathology	CCH	19 (30%)
	MTC	44 (70%)
Size MTC (mm)		4 (3.5)

F = female; M = male; Pre-op Ct = preoperative serum basal calcitonin, expressed as a ratio (Ct value/upper limit of the normal range value); MOD-risk = ATA moderate risk mutational profile; H-risk = ATA high risk mutational profile; TT = total thyroidectomy; CNR = central neck lymph node removal; CCH = C-cells hyperplasia; MTC = medullary thyroid carcinoma.

**Table 2:** Clinical, pathological findings in 63 thyroidectomized patients according to age at surgery.

Characteristics		Pediatric	Adults	*p*-Value
		(n = 21)	(n = 42)	
Age at surgery (yrs)		12 (6.5)	31 (21)	<0.0001
Gender	Female	12 (57%)	27 (64%)	0.58
	Male	9 (43%)	15 (36%)	
Pre-op Ct levels		0.6 (0.8)	1.15 (4.62)	0.04
Pre-op Ct levels	<1	14 (67%)	17 (40%)	0.06
	≥1	7 (33%)	25 (60%)	
*RET* mutation	MOD-risk	14 (67%)	34 (81%)	0.23
	H-risk	7 (33%)	8 (19%)	
Surgery	TT	13 (62%)	14 (33%)	0.057
	TT + CNR	8 (38%)	28 (67%)	
Pathology	CCH	11 (52%)	8 (19%)	0.009
	MTC	10 (48%)	34 (81%)	
Size MTC (mm)		2 (2.5)	5 (4)	0.0016

Pre-op Ct = preoperative serum basal calcitonin, expressed as a ratio (Ct value/upper limit of the normal range value); MOD-risk = ATA moderate risk mutational profile; H-risk = ATA high risk mutational profile; TT = total thyroidectomy; CNR = central neck lymph node removal; CCH = C-cells hyperplasia; MTC = medullary thyroid carcinoma.

**Table 3:** Clinical and pathological findings according to the ATA risk levels.

Characteristics		All Patients	MOD-Risk	HIGH-Risk	*p*-Value
		n = 63	(n = 48)	(n = 15)	
Sex ratio (Female/Male)		39/24	31/17	8/7	0.54
Age at surgery	<18	21	14 (29%)	7 (47%)	0.23
	≥18	42	34 (71%)	8 (53%)	
Pre-op Ct levels	<1	31	26 (54%)	5 (33%)	0.23
	≥1	32	22 (46%)	10 (66.7%)	
Surgery	TT	27	23 (48%)	4 (27%)	0.23
	TT + CNR	36	25 (52%)	11 (73%)	
Pathology	CCH	19	18 (37.5%)	1 (7%)	0.026
	MTC	44	30 (62.5%)	14 (93%)	

Pre-op Ct = preoperative serum basal calcitonin, expressed as a ratio (Ct value/upper limit of the normal range value); MOD-risk = ATA moderate risk mutational profile; H-risk = ATA high risk mutational profile; TT = total thyroidectomy; CNR = central neck lymph node removal; CCH = C-cells hyperplasia; MTC = medullary thyroid carcinoma.

**Table 4:** Morbidity related to prophylactic and early thyroidectomy in pediatric and adult population.

Characteristics		Pediatric	Adults	*p*-Value
		(n = 21)	(n = 42)	
Overall Morbidity	yes	7 (33%)	13 (31%)	>0.99
	no	14 (67%)	29 (69%)	
Hypoparathyroidism	Transient	7 (33%)	11 (26%)	0.57
	Permanent	0	4 (10%)	0.29
Vocal cord paresis	Transient	1 (5%)	3 (7%)	>0.99
	Permanent	1 (5%)	1 (2%)	>0.99

**Table 5:** Predictive factors of MTC in 63 patients undergoing prophylactic and early thyroidectomy.

Characteristics		All Patients	Univariate Analysis			Multivariate Analysis			
			CCH	MTC	*p*-Value	Odds Ratio	95% CI	*p*-Value	
			n = 19	n = 44					
Age at surgery	<18	21	11 (58%)	10 (23%)	0.01	7.51	1.72–32.8		0.007
	≥18	42	8 (42%)	34 (77%)					
Gender	Female	39	12 (63%)	27 (61%)	0.89				
	Male	24	7 (37%)	17 (39%)					
Pre-op Ct levels	<1	31	15 (79%)	16 (36%)	0.002	5.02	1.22–20.6		0.02
	≥1	32	4 (21%)	28 (64%)					
*RET* mutational risk	MOD	48	18 (95%)	30 (68%)	0.026	18.5	1.63–209		0.01
	H	15	1 (5%)	14 (32%)					

Pre-op Ct = preoperative serum basal calcitonin, expressed as a ratio (Ct value/upper limit of the normal range value); MOD-risk = ATA moderate risk mutational profile; H-risk = ATA high risk mutational profile; TT = total thyroidectomy; CCH = C-cells hyperplasia; MTC = medullary thyroid carcinoma.

## Data Availability

Some or all datasets generated during and/or analyzed during the current study are not publicly available but are available from the corresponding author on reasonable request.
